# Relationship between Motor Competence, Physical Fitness, and Academic Achievement in Young School-Aged Children

**DOI:** 10.1155/2021/6631365

**Published:** 2021-02-05

**Authors:** Maja Batez, Živan Milošević, Ivan Mikulić, Goran Sporiš, Draženka Mačak, Nebojša Trajković

**Affiliations:** ^1^Faculty of Sport and Physical Education, University of Novi Sad, Serbia; ^2^Sports Diagnostic Center, Šabac, Serbia; ^3^Faculty of Kinesiology, University of Zagreb, Croatia; ^4^Faculty of Sport and Physical Education, University of Niš, Serbia

## Abstract

Children in schools are facing many academic challenges. Moreover, there is constant pressure on children and parents to maximize academic achievement. We aimed to determine the relationship between motor competence, physical fitness, and academic achievement in young school-aged children. Participants were 130 elementary school children (mean ± SD8.60 ± 0.61 years; 51 boys and 79 girls) from Serbia. The KTK (Körperkoordinations Test für Kinder) battery of tests was used to assess the motor competence in children; children' physical fitness was assessed using the EUROFIT battery of tests, while academic achievement was assessed based on the children's GPA (grade point average) scores at the end of the school year. Pearson's *r* showed the weak to moderate relationships between the GPA and motor competence and physical fitness measures. The GPA correlates positively and significantly with almost all motor competence and physical fitness measures, but negatively with BMI (*p* ≤ 0.05). However, the hierarchical linear regression indicated only the plate tapping and sit and reach as the significant predictors of the GPA. Although both tests positively affect the GPA, the plate tapping (*B* = −0.22, *p* = 0.02) tends to influence the GPA more than the sit and reach test (*B* = 0.18, *p* = 0.04) after adjusting for effects of motor competence (*B* = 0.19, *p* = 0.03), age (*B* = −0.01, *p* = 0.89), and BMI (*B* = −0.19, *p* = 0.03). This study provides evidence demonstrating that academic achievement is generally associated with physical fitness and motor competence in children. However, plate taping and sit and reach were accounted as the most important predictors for academic achievement.

## 1. Introduction

Motor development and cognitive development have been studied separately, and they have generally been viewed as independent phenomena, although occurring in the same organism over the same time period. However, recently, motor development and cognitive development showed to be much more interrelated than what has been previously stated [1]. Recent studies have increasingly indicated the positive effects of motor competence and physical fitness on health status in children [2–5]. Moreover, the strength of relationship between motor competence and health status tends to increase from childhood to adolescence [6]. Most of the research on children's academic achievement has been focusing on physical activity showing the beneficial effects on academic performance [7–10]. Having in mind that the development of motor skill competence is a primary underlying mechanism that promotes engagement in physical activity and fitness [11], we think that similar relationship between motor competence, physical fitness, and academic achievement was logical. However, studies that examined the association between physical activity, motor competence and physical fitness, and academic achievement showed contradictory results, especially in the directionality of association [12]. Apart from this well-known effect, a large number of authors produced research on the effect of the level of motor skills on the cognitive abilities and academic achievement in the children.

The results of the study by Abdelkarim et al. [13] lead to a conclusion that encouraging motor competence in the prepubescent children during their elementary school years is associated with better academic achievement. This was confirmed by several other studies indicating the positive effects of motor competence on academic achievement [7, 12, 14–20]. Regarding physical fitness, Sardinha et al. [21] showed in their longitudinal study that an improvement in fitness in children increases the probability of higher academic achievement. This was confirmed by a recent systematic review by Donnelly et al. [9]. However, the authors stated that studies examining the associations among fitness and academic achievement showed inconsistent findings and that numerous elements of fitness are still unexplored.

Highly demanding curriculum requirements that schools, teachers, and parents put before children since the very start of their elementary education do not leave much time for exercise and improvement of fitness. In Serbia, it is obligatory to have two classes (2 × 45 min) of physical education per week, which is not sufficient enough to accomplish high level of motor competence and physical fitness. A possible relationship with academic achievement would put significantly more impact on exercise during their stay in school. Therefore, it is necessary to point out the importance of motor competence and physical fitness in younger children due to inconsistent findings [14] concerning the connection and the contribution to better academic achievement. Moreover, it is not defined which distinct academic domains are connected with physical fitness and academic achievement. Accordingly, the purpose of this paper is to examine the relationship between motor competence, physical fitness, and academic achievement in young school-age children.

## 2. Materials and Methods

### 2.1. Subject

This cross-sectional study included 130 elementary school children (Table 1). All children attended a standard school program in primary school from the territory of the city of Sabac (urban area), Republic of Serbia. We randomly selected four elementary schools and one class within each school. All students and their parents consented to participate in the study. The inclusion criteria were that children were healthy without any musculoskeletal injury or diseases. Ethical approval was obtained from the University Ethics Board at the Faculty of Sport and Physical Education in Novi Sad (reference no. 46-10-06/2020-1).

### 2.2. Procedures

All assessments were performed in accordance with the ethical standards laid down in the Declaration of Helsinki. The assessments included body height and weight, academic achievement, the Körperkoordinations Test für Kinder, and the Eurofit Physical Fitness Test Battery test. All assessments were conducted as part of the project “Bring sports to schools-Grow healthy” which is approved by the Serbian Ministry of Education, Science, and Technological Development (ref. no. 601-00-54/2012-15), which is under implementation on the territory of the city of Sabac (Republic of Serbia). The members of the “Sports Diagnostic Center of Sabac,” professors of physical education and sports sciences, assessed measures of physical fitness and motor competence. The Körperkoordinations Test für Kinder and the Eurofit Physical Fitness Test Battery tests were performed in the school gyms in the form of circuit exercises.

The participants were informed about the purpose and the technique of the tests and were given clear instructions on how to do the tests precisely, quickly, and consistently, in accordance with the factor being measured. Each test item was accompanied by specific instructions orally presented to each of the participants. Trials were allowed for each test item to allow for a familiarization with the tests. Following the familiarization, the participants performed the tests and the corresponding results were recorded.

### 2.3. Academic Achievement

The data on the children's grade point average (GPA) scores at the end of the school year were taken from the school records upon approval by the elementary schools' principals. We accounted two mandatory subjects for the GPA, the Serbian language and mathematics. According to the rulebook [22] on student assessment in Serbian schools, the grades are numerical. The student was evaluated on the basis of oral examination of achievement, written examination of achievement, and practical work and in accordance with the course program. Numerical grades are as follows: outstanding (5)—a student who achieves outstanding progress in mastering the subject program and completely independently fulfilling the requirements; very good (4)—a student who achieves significant progress in mastering the subject program and completely independently fulfilling the requirements; good (3)—a student who achieves progress in mastering the subject program and completely independently fulfilling the requirements; acceptable (2)—a student who achieves minimal progress in mastering the subject program and fulfilling with the help of the teacher the requirements that are determined in most of the basic level of achievement; and insufficient (1)—a student who does not achieve minimal progress in mastering the subject program and does not meet the requirements even with the help of the teacher.

The students must have four grades during one semester, in order to determine their final grade. The final grade is as follows: outstanding (5), if the arithmetic mean of all individual grades is at least 4.50; very good (4), if the arithmetic mean of all individual grades is from 3.50 to 4.49; good (3), if the arithmetic mean of all individual grades is from 2.50 to 3.49; acceptable (2), if the arithmetic mean of all individual grades is from 1.50 to 2.49; and insufficient (1), if the arithmetic mean of all individual grades is less than 1.50.

### 2.4. Motor Competence

The Körperkoordinations Test für Kinder (KTK) battery test was used to assess the level of motor competence [23]. The KTK battery consists of four test items: (1) walking backwards three times along three different wooden beams (a maximum of 72 steps), (2) single-leg hopping over a 5 cm high foam obstacle (a maximum of 78 points for both legs), (3) lateral jumping over a low obstacle within a 15-second time frame two times (a sum of jumps over two trials), and (4) lateral movement across the floor using 2 wooden platforms for 20 seconds (a sum of relocations over two trials). The raw performance score of each test item was converted into a standardized motor quotient (MQ) adjusted for age and gender according to the normative data tables. Likewise, the sum of all four item MQs was transformed into an MQ total. The battery of tests was customized for 5- to 15-year-olds, with high reliability (reliability coefficient from 0.90 to 0.97 for the total battery of tests) and validity (*r* = 0.60-0.80 for the intercorrelation of KTK subtests) [24, 25].

### 2.5. Physical Fitness

The range of children's motor skills was assessed using the Eurofit Physical Fitness Test (EUROFIT) battery tests [26]. Each item test was performed two times, and a better result was recorded. A plate tapping test was used to estimate the reaction rate. The subject moves the preferred hand back and forth between the discs (which placed with their centers 60 cm apart on the table) over the hand in the middle as quickly as possible. This action is repeated for 25 full cycles (50 taps). The time taken to complete 25 cycles is recorded. The test was performed twice, and the best result is recorded. A standing long jump test was used to estimate the explosive leg power. The subject stands behind a line marked on the ground with feet slightly apart. The subject attempts to jump as far as possible, landing on both feet without falling backwards. The measurement is taken from the take-off line to the nearest point of contact on the landing (back of the heels). The “sit-ups in 30 seconds” test was used to estimate the abdominal and hip flexor muscles. On the command “Go,” the subject raises the chest so that the upper body is vertical and then returns to the floor. For each sit-up, the back must return to touch the floor. The maximum number of correctly performed sit-ups in 30 seconds is recorded. A 10 × 5 m shuttle run test was used to estimate the speed and agility. When instructed by the timer, the subject runs to the opposite marker, turns, and returns to the starting line. This is repeated five times without stopping (covering 50 meters in total). At each marker, both feet must fully cross the line. Record the total time taken to complete the 50 m course.

### 2.6. Data Analysis

Statistical analysis was carried out using SPSS (v19.0, SPSS Inc., Chicago, IL, USA), and data are presented as mean ± SD unless otherwise stated. Data were log-transformed analysed but reported raw for the sake of clarity when the Kolmogorov–Smirnov test failed to confirm normality.

The *t*-test for independent samples analysed the mean differences between boys and girls in the study outcomes. If Levene's test did not assume equal variances, we used the *t*-test with a Satterthwaite approximation for the degrees of freedom. By the visual inspection of a residual scatter plot, we confirmed homoscedasticity.

Pearson's correlation coefficients (*r*) were used to investigate associations among motor competence, physical fitness, and academic achievement. The degrees of statistically relevant Pearson's correlation are defined in the bivariate linear relationship as weak (±0.10), moderate (±0.30), and strong (±0.50) [27]. The correlation matrix was inspected to assess multicollinearity (*r* ≥ 0.8) [28].

Finally, we modelled a GPA using a hierarchical linear regression building approach. Hence, three sequential linear regression models evaluated a relative influence (*B*, standardized coefficient) of motor competence (block 2) and physical fitness measures (block 3) on an academic achievement, while controlling for age and BMI effects (block 1). Overall fit of the academic achievement model in the function of motor competence and physical fitness measures (adjusted for age and BMI) is represented by the coefficient of determination (*R*^2^) and adjusted coefficient of determination (adjusted *R*^2^). We compared the models using change statistics (in *R*^2^ and *F*) from block 1 to block 2 and from block 2 to block 3. The level of significance was set at *p* ≤ 0.05.

## 3. Results

Table 2 presents motor competence and physical fitness for the pooled sample and by gender. The *t*-test for independent samples showed no significant differences among boys and girls in the GPA (*t*_(144)_ = −0.65, *p* = 0.51), the KTK MQ (*t*_(144)_ = −1.44, *p* = 0.15), plate tapping (*t*_(144)_ = −0.94, *p* = 0.35), standing broad jump (*t*_(96.93)_ = −0.92, *p* = 0.36), sit-ups (*t*_(92.17)_ = 0.94, *p* = 0.71), 10 × 5 m shuttle run (*t*_(144)_ = −1.16, *p* = 0.25), and sit and reach (*t*_(144)_ = −0.95, *p* = 0.35).

The correlation matrix of Pearson's *r* showed the weak to moderate relationships between the GPA and motor competence and physical fitness measures. The GPA correlates positively and significantly with almost all motor competence and physical fitness measures, but negatively with BMI (*p* ≤ 0.05). No multicollinearity was observed among the predictors. See Table 3 for the results from bivariate analysis.

A hierarchical linear regression yields that the full model of academic achievement was significant (block 3). The inclusion of the KTK MQ significantly improved the prediction of the GPA (change statistics: *R*^2^ = 0.03, *F*_(1, 142)_ = 4.81, *p* = 0.03) over and above age and BMI (block 1). However, the addition of physical fitness measures did not significantly enhance the prediction of the GPA (change statistics: *R*^2^ = 0.06, *F*_(5, 137)_ = 1.85, *p* = 0.11), although the plate tapping and sit and reach significantly influenced the GPA. The plate tapping tended to influence the GPA more than the sit and reach test. The full model of GPA is shown in Table 4.

## 4. Discussion

The aim of the study was to examine the relation between children's final GPA scores at the end of the school year and the results achieved on motor competence and physical fitness tests. The main findings of the present study are weak positive correlations between motor competence, physical fitness, and academic achievement (*r* from 0.14 to 0.22). The linear regression models showed positive association only for plate tapping (*B* = −0.22, *p* = 0.02) and sit and reach (*B* = 0.18, *p* = 0.04) after excluding the KTK MQ influence and adjusting for age and BMI effects. The obtained results indicate that gender was not the important predictor in this age group, showing the similar results for boys and girls in GPA (*p* = 0.51), the KTK MQ (*p* = 0.15), plate tapping (*p* = 0.35), standing broad jump (*p* = 0.36), sit-ups (*p* = 0.71), 10 × 5 m shuttle run (*p* = 0.25), and sit and reach (*p* = 0.35). As expected, the body mass index was negatively associated with GPA scores (*B* = −0.17, *p* = 0.05) in children.

There is sufficient evidence that children with low motor competence have a higher probability of having low academic achievement [17, 29–31]. The results of the present study indicate that motor development is positively related to successful academic achievement, supporting previously provided evidence about positive relationship between cognitive and motor developmental trajectories [1, 32–34]. Accordingly, highly developed motor abilities may facilitate cognitive function in children and thus contribute to better academic achievement, which is confirmed by other studies [31, 35]. Moreover, coordinative exercises are involved in the activation of the cerebellum, influencing motor functions [36] and working memory [37] as well as attention [38]. In the end, better motor competence leads to better overall health, as has been shown in the case of physical fitness [39], which may further contribute positively to academic achievement [17]. However, after adjusting the motor competence influence on the GPA for BMI effect, regression analysis showed that KTK MQ could not significantly predict academic achievement. This could be due to smaller sample compared to other studies that examined the influence of motor competence on academic achievement. Moreover, the children in the present study showed very low level of KTK MQ (85.13), which could impact the association between motor competence and academic achievement. A recent study [40] confirmed that MQ KTK is actually stagnating (1.1%) or showing a delayed development (14.7%) of motor competence opposite to the reference sample [41]. Moreover, the same study showed statistically significant variability in trajectories of change in motor competence in individual children. Chagas et al. [29] in their recent study showed no significant associations between motor coordination and academic achievement in boys and girls. Therefore, longitudinal and prospective studies are needed in order to determine how better or worse motor competence can influence the academic performance of children throughout schooling.

By analysing the relationship between academic achievement and physical fitness, significant correlations were observed in the plate tapping test, standing long jump, and sit and reach test. However, the linear regression model showed positive association only for plate taping and sit and reach. Studies examining similar subjects also indicate a positive correlation between the plate tapping test and academic achievement [42]. According to Fernandes et al. [42], action systems [43] and anticipatory systems [44] involve different brain regions related to attentional control and visual processing and specific brain regions related to response selection and planning [45]. Moreover, it was stated that the deficit in visuospatial attention is related to motor and cognitive deficits found in children with developmental coordination disorder [46]. Altogether, the above-mentioned fact points to the importance of coordinative moves during plate tapping and accordingly to faster processing and longer retention of information during school. A positive correlation between academic achievement and fitness tests indicates that physically fit children have better academic performance. Most of the studies regarding physical fitness examined the association between aerobic fitness and academic achievement. A recent systematic review demonstrated positive relations between academic performance and aerobic fitness [9]. Results regarding relationship between the sit and reach test and academic achievement are inconsistent [20, 47]. Castelli et al. [20] found that flexibility was not related to general academic achievement, reading, and mathematics in third- and fifth-grade students. On the contrary, there was a positive correlation between sit and reach and mathematics scores [47]. The possible explanation could be because the association between fitness (sit and reach) and academic achievement may reflect the achievement orientation of motivated students [39]. Additionally, children's physical fitness may lead to a better overall health which can contribute positively to academic achievement [39]. Poor physical fitness at an early age may contribute to a negative path of academic development as indicated by Kantomaa et al. [48]. Several authors tried to give reasonable explanations regarding significant associations between physical fitness and academic achievement in children. However, the complexity of fitness and academic achievement relationship makes it difficult to specifically identify possible mechanisms. Hillman et al. [49] stated that fitter children have a more effective neuroelectric profile than less fit children on a stimulus discrimination task. Moreover, the motivation could have significant contribution for this significant relationship because children who perform better and enjoy in school may be more likely to exert more effort on academic tests and physical fitness [20]. In the end, weight status (BMI) was inversely associated with GPA scores in the current study. However, it could be speculated that the effect of weight status on academic achievement could be mediated by motor competence or physical fitness which was stated earlier [50]. This study did not comprise a large sample of participants, which may be deemed one of the drawbacks of the same. On the other hand, this study incorporated field tests where the motivation of the children played a significant role, in comparison with the laboratory tests which tend to produce more accurate results. It is safe to assume that inclusion of parameters such as parents' economic status, physical activity questionnaire, and availability of sport facilities near the place of residence would allow for a more comprehensive picture of the relationship between the motor skills of prepubescent children and their performance at school. Moreover, the motor competence and physical fitness influence on academic achievement was not adjusted for the intellectual ability effects. Future research should provide more evidence on the individual relationship of each subject to motor skills in order to discuss the causal relationships in more detail.

## 5. Conclusions

This study provides evidence demonstrating that academic achievement is generally associated with physical fitness and motor competence in children. However, when accounting motor competence and physical fitness in the regression model, plate tapping and sit and reach were the only significant predictors for academic achievement. More research is needed to fully understand the relationship between motor competence, physical fitness, and cognition in children and possible mediators that could influence the academic achievement in children. Nevertheless, this type of studies may help all the subjects involved in the process of education to recognize, understand, and design ways of facilitating creative and stimulating physical exercises both in and out of school. This primarily refers to creative and challenging motor tasks in the course of sensitive periods that may contribute to the development of self-confidence in children, improvement of memory, anticipation, and all other qualities that may result in better academic achievement.

## Figures and Tables

**Table 1:** Sample characteristics.

	Total	Boys (*N* = 51)	Girls (*N* = 79)
Age (years)	8.60 ± 0.61	8.60 ± 0.63	8.59 ± 0.60
Height (cm)	138 ± 0.08	139 ± 0.08	137 ± 0.08
Weight (kg)	33.43 ± 8.53	34.50 ± 8.36	32.32 ± 9.15
BMI (kg/m^2^)	17.32 ± 3.29	17.81 ± 3.30	17.04 ± 3.49

Values are mean ± SD. BMI: body mass index.

**Table 2:** Sample characteristics and study outcomes in the pooled sample and among boys and girls.

	Total	Boys (*N* = 51)	Girls (*N* = 79)
GPA	4.64 ± 0.71	4.58 ± 0.56	4.72 ± 0.65
KTK MQ	85.13 ± 19.15	80.39 ± 16.69	86.10 ± 18.34
Plate tapping (s)^¥^	16.00 ± 2.56	16.43 ± 2.86	16.06 ± 2.31
Standing long jump (cm)	124.79 ± 25.21	124.73 ± 28.55	120.96 ± 21.28
Sit-ups (frequency)	17.03 ± 5.23	17.00 ± 6.33	16.67 ± 4.61
10 × 5 m shuttle run (s)^¥^	25.62 ± 3.07	25.39 ± 3.55	25.94 ± 2.84
Sit and reach (cm)	17.79 ± 6.28	18.47 ± 6.62	17.30 ± 6.01

Values are mean ± SD. ^¥^ Reverse scoring. GPA: grade point average; KTK MQ: motor quotient from the Körperkoordinations Test für Kinder.

**Table 3:** The correlation matrix showing Pearson's *r* for the motor competence, physical fitness, and academic achievement.

	GPA	Age	BMI	KTK MQ	Plate tapping (s)^¥^	Standing broad jump (cm)	Sit-ups (frequency)	10 × 5 m shuttle run (s)^¥^
Age	-0.01							
BMI	-0.19^∗^	-0.03						
KTK MQ	0.22^∗∗^	-0.17^∗^	-0.21^∗∗^					
Plate tapping (s)^¥^	-0.20^∗∗^	0.35^∗∗^	0.08	-0.26^∗∗^				
Standing long jump (cm)	0.15^∗^	-0.43^∗∗^	-0.28^∗∗^	0.48^∗∗^	-0.34^∗∗^			
Sit-ups (frequency)	0.09	-0.13	-0.25^∗∗^	0.19^∗^	-0.40^∗∗^	0.46^∗∗^		
10 × 5 m shuttle run (s)^¥^	-0.06	0.25^∗∗^	0.19^∗^	-0.37^∗∗^	0.24^∗∗^	-0.56^∗∗^	-0.48^∗∗^	
Sit and reach (cm)	0.14^∗^	-0.03	0.10	0.08	0.15^∗^	-0.07	-0.14^∗^	-0.09

^¥^Reverse scoring. GPA: grade point average; BMI: body mass index; KTK MQ: motor quotient from the Körperkoordinations Test für Kinder.

**Table 4:** Linear regression models of academic achievement.

Predictors	GPA					
	Block 1		Block 2		Block 3	
	*B*	*p*	*B*	*p*	*B*	*p*
Age	-0.01	0.89	0.02	0.80	0.10	0.29
BMI (kg/m^2^)	-0.19	0.03	-0.15	0.08	-0.16	0.07
KTK MQ			0.19	0.03	0.12	0.20
Plate tapping (s)^¥^					-0.22	0.02
Standing long jump (cm)					0.09	0.47
Sit-ups (frequency)					-0.02	0.85
10 × 5 m shuttle run (s)^¥^					0.10	0.36
Sit and reach (cm)					0.18	0.04
*R* ^2^	0.04		0.07		0.13	
Adjusted *R*^2^	0.02		0.05		0.07	
*F*	2.38		3.37^∗^		2.46^∗^	

1 = males; 2 = females. ^¥^Reverse scoring. GPA: grade point average; BMI: body mass index; KTK MQ: motor quotient from the Körperkoordinations Test für Kinder; *B*: standardized coefficient; *R*^2^: coefficient of determination; *F*: *F* statistic. ^∗^Significant at *p* ≤ 0.05.

## Data Availability

The datasets analysed during the current study are available from the corresponding author on reasonable request.
